# A comparison of the pharmacokinetic and pharmacodynamic properties of nitroglycerin according to the composition of the administration set: A preliminary study

**DOI:** 10.1097/MD.0000000000009829

**Published:** 2018-03-02

**Authors:** Choon Ok Kim, Jeongyun Song, Ji Young Min, Su Jung Park, Hye Mi Lee, Hyo-Jin Byon

**Affiliations:** a Department of Clinical Pharmacology and Clinical Trials Center, Severance Hospital, Yonsei University Health System, Seodaemun-gu, Seoul; b Department of Anesthesiology and Pain Medicine, Yeoncheon Public Health Center, Yeoncheon-gun, Gyeonggi-do; c Department of Anesthesiology and Pain Medicine; d Anesthesia and Pain Research Institute, Yonsei University College of Medicine, Seodaemun-gu, Seoul, Republic of Korea.

**Keywords:** administration set, drug sorption, nitroglycerin, polyolefin

## Abstract

**Background:**

There is a risk of drug sorption into an intravenous administration set composed of polyvinyl chloride (PVC), polyurethane (PU), or polyolefin (PO). This has implications on the dose of the active ingredient the patient receives, and thus therapeutic success. This study aimed to determine the plasma concentration of nitroglycerin and the effect of nitroglycerin on patients based on the composition of the administration set.

**Methods:**

Using a randomized, open-labeled, 3 × 3 crossover method, 9 volunteers were assigned to 3 groups. In period I, nitroglycerin (100 μg/mL) was infused via a PVC- (group A), PU- (group B), or PO-based (group C) administration set. In period II, PU- (group A), PO- (group B), and PVC-based (group C) administration sets were used, and in period III, PO- (group A), PVC- (group B), and PU-based (group C) administration sets were used. The rate of drug administration in all periods was 12 mL/hour for 30 minutes using an infusion pump. Blood samples were collected, and the plasma concentrations of nitroglycerin were analyzed using validated high-performance liquid chromatography coupled with tandem mass spectrometry. Blood pressure was determined using a sphygmomanometer applied to the other upper arm at an interval of 5 minutes.

**Results:**

We observed that the mean plasma concentration of nitroglycerin over time when administered using a PO-based tube was higher than that when using a PU- or PVC-based tube. When the percent change of the mean arterial pressure from baseline at each time point was compared among groups, there were statistically significant differences between PU and PO or PVC at most points during nitroglycerin infusion.

**Conclusion:**

Our results showed higher nitroglycerin plasma concentration and lower arterial pressure when a PO-based administration set was used than when a PVC- or PU-based administration set was used. PO-based administration sets may be more appropriate for nitroglycerin administration compared to those composed of PVC or PU.

## 1 Introduction

The risk of drug sorption into the administration set has been well documented.^[1–3]^ Drug sorption can influence the prognosis of patients by preventing them from receiving the physician-intended drug dosage. Nitroglycerin is a nitrate used for vasodilation in patients with hypertension and ischemic heart disease.^[4]^ Sorption of nitroglycerin into the administration set can increase the risk of cardiovascular instability due to the administration of an inadequate dosage. In previous studies, the possibility of nitroglycerin sorption into administration set tubes composed of polyvinyl chloride (PVC), polyurethane (PU), or polyolefin (PO) has been discussed.^[5,6]^ Park et al^[7]^ showed PO administration set prohibited drug sorption when compared to the PVC and PU administration set; however, these were in vitro studies. Altavela et al^[8]^ also revealed that patients who received intravenous nitroglycerin through a PVC administration set had the same clinical response as patients given the drug through a polyethylene set. There is a paucity of in vivo studies focused on the plasma concentration and efficacy of drugs comparing PO, PU, and PVC administration sets. As individual variation among patients can affect sensitivity to drugs, and this could in turn influence therapeutic outcomes, we cannot ascertain whether drug sorption into the administration set contributes to differences in a drug's effect. Moreover, the clinical environment is different from that of a laboratory in terms of the total length of the intravenous set, means of connecting the set, and drug infusion rate. The present study aimed to explore the effect of the composition of the administration set on the plasma concentration and effect of nitroglycerin on patients.

## 2 Methods

### 2.1 Chemicals

We used 3 different types of administration sets with tubes composed of PVC, PU, and PO. Di-(2-ethylhexyl) terephthalate (DEHT) was used as the plasticizer for the PVC tube. All tubes had a length of 100 cm, and were manufactured and supplied by Polyscientech Co., Ltd (Anseong, Gyeonggi, Korea). An independent experimenter diluted nitroglycerin (Bayer Pharma AG, Leverkusen, Germany) with 90 mL of normal saline to obtain a final concentration of 100 μg/mL.

### 2.2 Patients

The Severance Hospital Institutional Review Board provided ethics approval for this study; we obtained written informed consent from all study participants. All volunteers were men, aged 19 to 50 years, with a BMI of 18.5 to 25 kg/m^2^. A complete medical history of all participants was obtained, and all of them underwent comprehensive physical screening and examination, EKG (electrocardiography), and laboratory tests. Exclusion criteria were as follows: a history of abnormalities of the cardiovascular, respiratory, renal, endocrinal, hepatic, hematologic, or gastrointestinal system or any other significant disease state that could affect pharmacokinetics (PK); drug addiction, including opioids, narcotics, or any other drugs, affecting the central nervous system; chronic use of any medication; and hypersensitivity to nitroglycerin or any other nitrate drugs. Nine volunteers were recruited to take part in the present study. The present study was a randomized, open-labeled, 3 × 3 crossover study; therefore, 3 volunteers each were randomly allocated to groups A, B, and C. In group A, a PVC-based tube was used in period I, and PU- and PO-based tubes were used in periods II and III, respectively. In group B, PU-, PO-, and PVC-based tubes were used in periods I, II, and III, respectively, and in group C, PO-, PVC-, and PU-based tubes were used in periods I, II, and III, respectively.

#### 2.2.1 Period I

On day 1, all volunteers fasted for 4 hours before arriving at the hospital. Water intake was restricted for an hour before and after the administration of nitroglycerin. Patients were subjected to EKG, noninvasive blood pressure monitoring, and pulse oximetry. A 23-gauge intravenous catheter was inserted into the forearm vein. Blood pressure was determined using an upper-arm sphygmomanometer at an interval of 5 minutes. The 100-cm-long administration set tube was connected to the catheter. Following predosing blood sample collection, nitroglycerin (100 μg/mL) was infused via PVC (group A)-, PU (group B)-, or PO (group C)-based tubes at the rate of 12 mL/hour for 30 minutes by using an infusion pump. Blood samples were collected at 2, 5, 10, 20, and 30 minutes after administration was initiated and at 3, 6, and 10 minutes after drug administration completed. A blood sample of 5 mL was transferred to an EDTA-containing tube and centrifuged within an hour after collection. The centrifuged plasma samples were placed on ice until they were transported to the laboratory for storage at −70°C. The plasma concentration of nitroglycerin was obtained from the laboratory within a month. Blood pressure and heart rate were recorded before administration and at 2, 4, 6, 8, 10, 14, 18, 22, 26, and 30 minutes after drug administration was initiated and at 4, 8, 12, 16, 20, 24, 28, and 32 minutes after drug administration was completed.

#### 2.2.2 Periods II and III

As there was a washout interval, period II was started 5 days or more after period I was concluded. The protocol used in period II for drug administration was the same as that in period I, except for the type of administration tube used in each group. Similarly, period III was initiated 5 days or more after period II was concluded. The summary of the intervention cycle is outlined in Table 1.

**Table 1:**
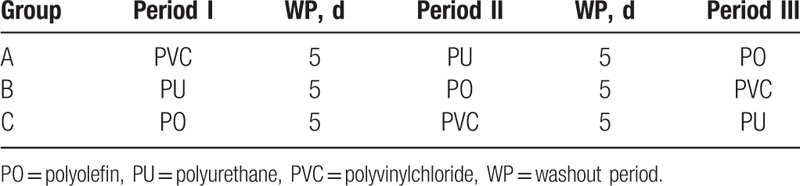
The summary of the intervention cycle.

### 2.3 Plasma nitroglycerin assay

The plasma concentrations of nitroglycerin were analyzed using validated high-performance liquid chromatography (HPLC, Agilent 1200 HPLC system; Agilent Technologies, Santa Clara, CA) coupled with tandem mass spectrometry (MS/MS, 4000Qtarp; ABSICX, Concord, Ontario, Canada). The analytical column was a Kinetex C18 (100 × 4.6 mm, 2.6 μm) (Phenomenex, Torrance, CA), and the mobile phase comprised solvent A (0.025 mM ammonium chloride) and solvent B (methanol). The separation method used was a gradient method. The organic solvent used was initially of low concentration (5% Solvent B), which was increased (95% Solvent B), and then decreased to the initial concentration (5% Solvent B). The total analysis time was 15 minutes; the flow rate was 550 μL/min. The working solutions of nitroglycerin were prepared at human plasma concentrations of 0.1 – 50.0 ng/mL. The sample was prepared as follows: an aliquot of 500 μL of human plasma was transferred to a polypropylene tube; 1000 μL of acetonitrile and the internal standard solution were added; and the mixture was vortex-mixed for a minute. The sample was centrifuged at 13,000 rpm for 10 minutes, and the supernatant was separated. A sample (15 μL) of the supernatant was then injected into an LC-MS/MS system. The concentrations of nitroglycerin were calculated from the calibration curve. The calibration standards showed acceptable linearity (correlation coefficient, *r*^2^ > 0.99) over a concentration range of 0.1 to 50.0 ng/mL, determined by a 1/*x*^2^ weighted least-squares linear regression analysis. The precision of the assay was less than 20%, and the accuracy was within the range of 80% to 120%.

### 2.4 PK analysis

The PK parameters of nitroglycerin were calculated via noncompartmental analysis using Phoenix 64 WinNonlin 7.0 software (Pharsight, Mountain View, CA). The maximum plasma concentration (*C*_max_) and the time to reach the *C*_max_ (*t*_max_) were determined directly from the observed data. The area under the plasma concentration–time curve from dosing time (0 hour) to the time of the last measurable concentration (AUC_last_) was calculated using the linear trapezoidal rule. Most of the samples collected after the infusion of nitroglycerin were below the limit of quantification, and the terminal elimination rate constant (λz), elimination half-life (*t*_1/2_), and the plasma clearance could not be calculated.

### 2.5 Safety assessment

Safety was assessed via physical examinations and by monitoring vital signs (systolic blood pressure, diastolic blood pressure, pulse rate, and body temperature), 12-lead ECG, and laboratory tests at predefined time points. In addition, adverse events (AEs) were evaluated throughout the study. Any undesirable sign, symptom, or medical condition occurring after the administration of the study drug was recorded, regardless of its suspected relationship to the study medication.

### 2.6 Statistical analysis

The PK data were analyzed and compared among the 3 administration sets (PO, PU, and PVC). All data are expressed as mean ± standard deviation (SD). The primary PK parameters (*C*_max_ and AUC_last_) were log-transformed and analyzed by analysis of variance (ANOVA) using a mixed-effects model. To compare the PK parameters, point estimates and 90% confidence intervals (CI) for the geometric mean ratios (PU/PO and PVC/PO) of the log-transformed *C*_max_ and AUC_last_ were also calculated. The repeated-measures mixed model was applied to compare the percent changes in mean arterial pressure (MAP, MAP = [SBP + 2DBP]/3) from baseline among the 3 administration set groups. The baseline MAP as a covariate and the administration set, time, period, sequence, and time-to-administration set interactions were analyzed as fixed effects. The demographic characteristics were analyzed using the Kruskal–Wallis test for comparison among the 3 sequences (A, B, and C). All analyses were conducted using SAS statistical software, version 9.2 (SAS Institute, Inc., Cary, NC). All statistical tests were 2-sided, and statistical significance was defined as *P* < .05.

## 3 Results

### 3.1 Study participants

**Figure 1:**
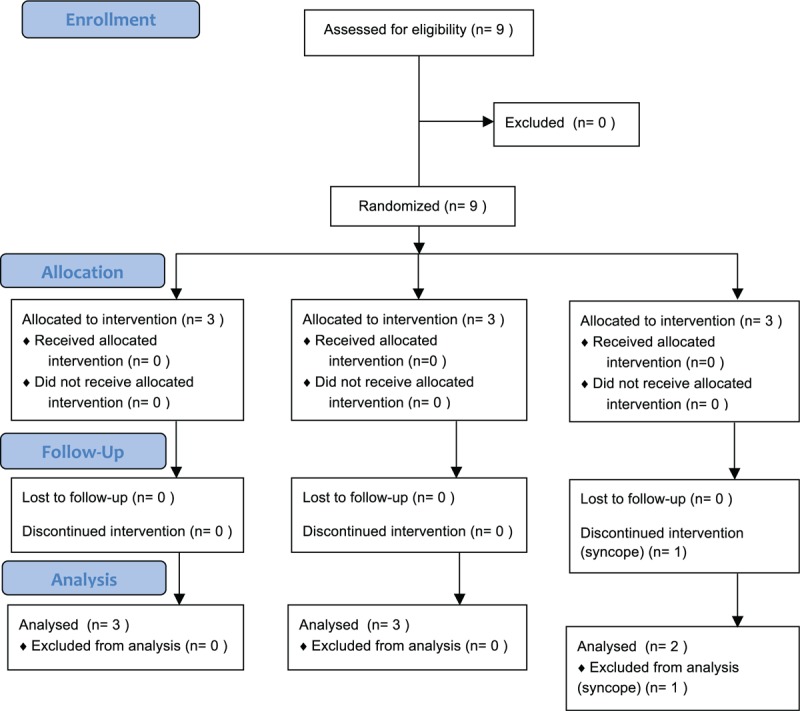
Study flow chart.

The progression of participants through the study is illustrated in Fig. 1. A total of 9 healthy Korean male participants were enrolled. Three participants were randomly assigned to each administration set sequence group. One participant in C group experienced syncope during nitroglycerin infusion with the PO administration set and was excluded from the study. All the other subjects completed the study. The participants in each sequence group were administered nitroglycerin intravenously for 30 minutes in the following sequence: PVC–PU–PO (A group), PU–PO–PVC (B group), and PO–PVC–PU (C group). There were no statistically significant differences in age, body weight, or body mass index among the sequence groups (*P* > .05, Table 2).

**Table 2:**
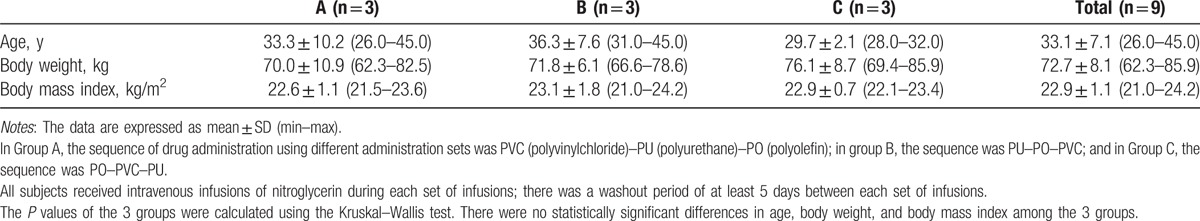
Demographics and baseline characteristics of the study population.

### 3.2 PK parameters

**Figure 2:**
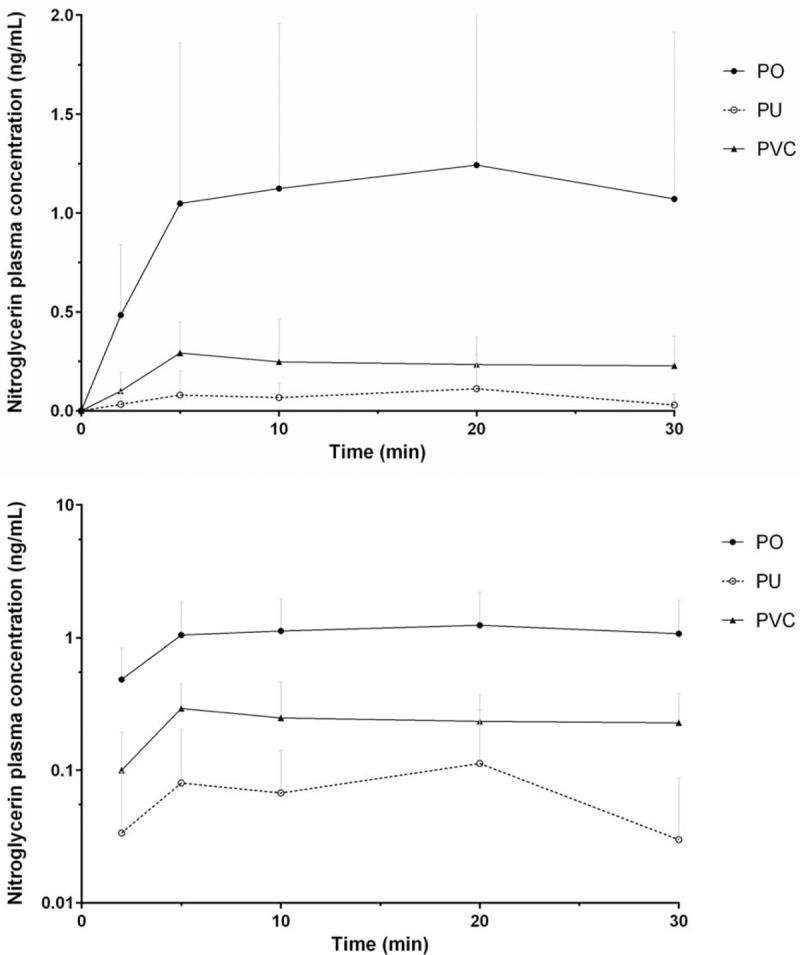
Mean plasma nitroglycerin concentration–time profiles after intravenous infusion in healthy male subjects (upper: linear scale, lower: semilogarithmic scale). PO = polyolefin, PU = polyurethane, PVC = polyvinylchloride.

The PK parameters were analyzed using data from the 8 subjects who completed this study. In all administration sets, plasma concentration of nitroglycerin increased rapidly after IV infusion and reached steady state after approximately 5 minutes (Fig. 2). When nitroglycerin was infused with the PO administration set, the mean plasma concentration of nitroglycerin over time was higher than that in the PU or PVC administration set group. The calculated PK parameters of nitroglycerin are shown in Table 3. The *C*_max_ and AUC_last_ of nitroglycerin in the PO or PVC administration set group were compared with those in the PU administration set group. When *C*_max_ and AUC_last_ of nitroglycerin in the PU administration set group were compared with those in the PO administration set group, the point estimates (with 90% CI) of the geometric mean ratios were 0.21 (0.07–0.65) and 0.14 (0.05–0.46), respectively. When *C*_max_ and AUC_last_ of nitroglycerin in the PVC administration set group were compared with those of the PO administration set group, the point estimates (with 90% CI) of the geometric mean ratios were 0.33 (0.17–0.65) and 0.31 (0.15–0.64), respectively.

**Table 3:**
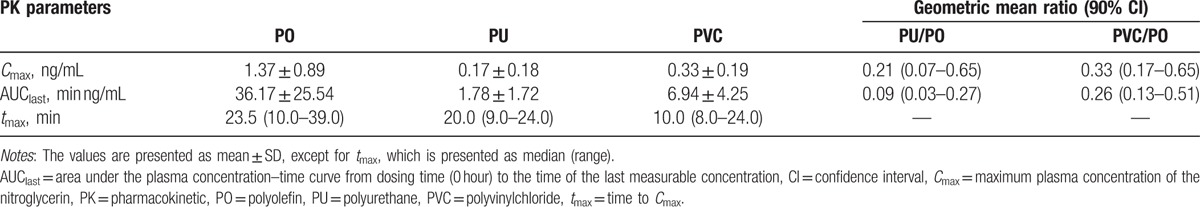
Pharmacokinetic parameters of nitroglycerin after intravenous infusion in healthy volunteers according to the administration set (n = 8).

### 3.3 Blood pressure

**Figure 3:**
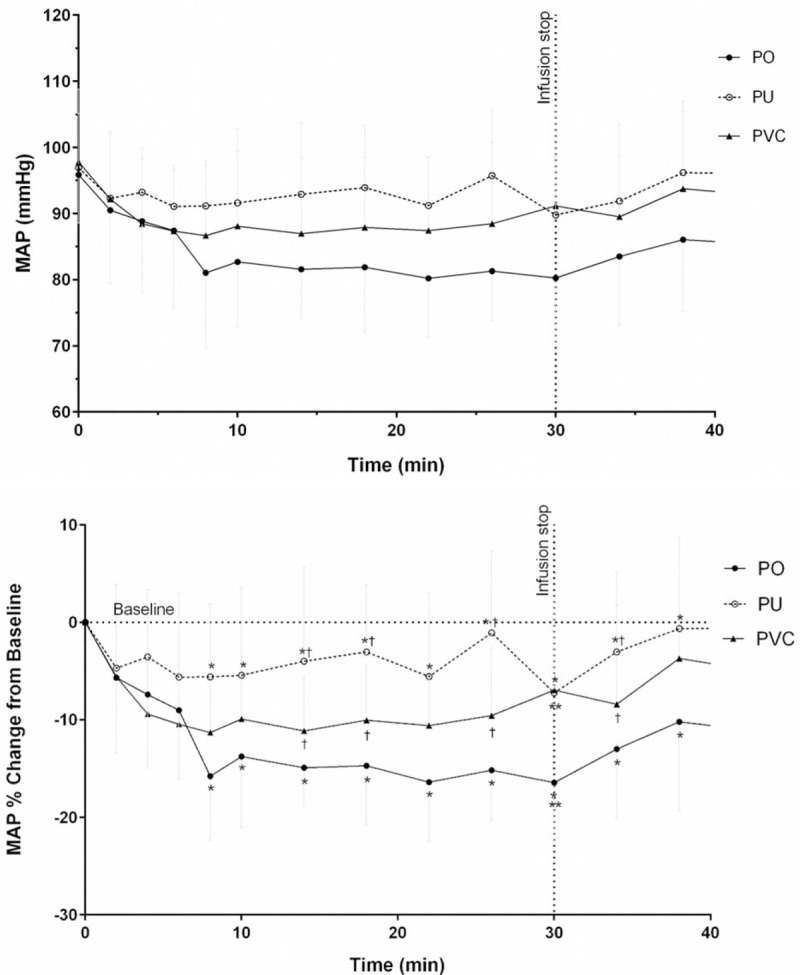
Mean (±standard deviation) arterial blood pressure after intravenous drug infusion in healthy male subjects. The marks mean *P* < .05, as compared between the PU and PO groups (∗), between the PU and PVC (^†^), and between the PO and PVC (∗∗) according to changes from baseline, by the repeated-measures mixed model. MAP = mean arterial pressure, PO = polyolefin, PU = polyurethane, PVC = polyvinylchloride.

Blood pressure of all but the one participant who was excluded was analyzed. When the percent change of MAP from baseline in each time point was compared among administration set groups, there were statistically significant differences between PU and PO or PVC administration set groups at most points during nitroglycerin infusion (*P* < .05, Fig. 3). There was no statistically significant difference between the PO and the PVC administration set groups in percentage change of MAP from baseline, except at the end of infusion.

### 3.4 Safety

There were 2 AEs in total, and they were considered related to the administration of nitroglycerin (Table 4). The first AE occurred during the infusion of nitroglycerin with the PO administration set; the subject developed a mild headache for 3 minutes, which resolved. This incidence did not occur in the other administration set groups. The second AE was syncope, which also occurred in the PO administration set group. In this case, blood pressure dropped rapidly right after the infusion of nitroglycerin, and the subject became unconscious for approximately 2 seconds. The subject recovered without any complications but was excluded from the study as a safety precaution. There were no life-threatening drug-induced AEs reported in this study.

**Table 4:**
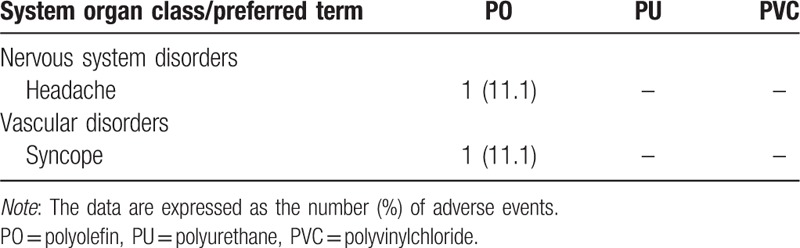
Incidence of adverse events per administration set.

## 4 Discussion

The material nature of a drug carrier can affect the delivery and effect of the drug. The sorption of a number of drugs from solutions by plastic bags was investigated.^[9]^ The developing drug carriers that facilitate the sustained and long-term release of drugs and improve the pharmacological bioavailability is an important issue.^[10,11]^ The material of an administration set can also influence on the effect of the drug.^[1,2]^

We performed a preliminary study to explore the effect of the materials used in different drug administration sets—namely, PVC, PU, and PO—on the PK and pharmacodynamic profile of nitroglycerin by comparing the plasma concentration and efficacy of nitroglycerin in the same subject. The *C*_max_, AUC_last_, and the effect of nitroglycerin on blood pressure decreased when PVC- and PU-based administration sets were used compared to those when the PO-based administration sets were used. This may be due to the sorption of nitroglycerin into the PVC- and PU-based administration sets.

Previous in vitro studies reported that the percentage sorption of nitroglycerin in PVC-based administration sets to be approximately 18% to 43% depending on the physiological condition.^[6]^ Nitroglycerin, however, has high intersubject pharmacokinetic variability, because it has extensive tissue distribution and rapid plasma clearance.^[4]^ Unfortunately, previous studies did not confirm whether the sorption of nitroglycerin into the administration sets made of different materials induced any clinically significant effect on the plasma concentration of nitroglycerin and blood pressure in patients. Thus, clinical studies such as the present study were required.

Our results showed that the mean plasma concentration of nitroglycerin in the PO administration set group over time was higher than those in the PU or PVC administration set group. These findings are in agreement with those of prior studies,^[9,12–14]^ suggesting that the tube material influences the nitroglycerin concentration due to drug sorption. This phenomenon can be explained by several theories. First, partitioning of drugs between the polymer and contact media is related to their hydrophobicity.^[15]^ Equilibrium of a drug between the tube material and the injectable media is affected by its partition coefficient, which is a major factor in drug sorption in the administration set. Drug sorption is also affected by the structure-based polarity and molecular weight of the polymers used in administration sets.^[16]^ Additionally, plasticizers may leach from a PVC-based tube, and the leaching of chemicals from the administration set can lead to a decrease in drug concentration.^[17–19]^

We also observed that the *C*_max_ and AUC_last_ of the PO group were higher than those of PVC and PU groups. These results indicated that the PO-based administration set could minimize the sorption of nitroglycerin into the tube. The possible explanation is that PO has a lower partition coefficient than does PVC or PU; therefore, a multicomponent polymer matrix (polyethylene elastomer, polypropylene elastomer, polybutadiene blend) of PO tubes can be equilibrated with reduced drug loss compared to PVC- and PU-based administration sets.^[20]^

In the present study, we noted that there were significant differences in lowering mean arterial pressure among the 3 types of tubes. The PO-based tube showed a more significant hypotensive effect than PU- and PVC-based tubes. The nitroglycerin acting on peripheral veins decreases venous return, resulting in a decrease in end-diastolic volume and pressure by the LaPlace relationship, and thus a decrease in myocardial oxygen consumption.^[21–23]^ The nitroglycerin also acts as a vasodilator of peripheral arteries. The decrease in mean arterial pressure would be expected to result in a further decrease in myocardial wall tension and an improvement in ventricular function.^[24,25]^ The administration of nitroglycerin intravenously through the PO-based tube may therefore allow more precise control of dosage and help avoid reduction in efficacy.

In this study, nitroglycerin was diluted to 100 μg/mL and infused at a rate of 12 mL/hour (20 μg/min) through a 100-cm tube, as per a recommended guideline for nitroglycerin infusion.^[4,8]^ Moreover, several studies revealed that as flow rate decreased or tube length increased, the amount of drug absorbed increased proportionately.^[26,27]^ Thus, caution should be exercised when applying our result to a different clinical setting.

There are several limitations to the present study. First, we did not measure the concentration of nitroglycerin in the tube. It is reasonable to assume, however, that sorption of nitroglycerin into the tube caused the difference in plasma nitroglycerin concentration and arterial pressure, according to the types of materials used in the tubes, as several in vitro studies on sorption of nitroglycerin into the tube have been carried out.^[5,6]^ A sampling of drug from the administration set can also interfere in measuring the plasma concentration and the effect of the drug. Second, we had a small sample size (9 volunteers). This study is preliminary; thus, further investigation with a larger sample size should be conducted in future. Third, we did not estimate the terminal elimination rate constant (λ_z_), elimination half-life (*t*_1/2_), or plasma drug clearance, because most of the samples collected after infusion of nitroglycerin were determined to be below the limit of quantification.

In conclusion, healthy volunteers administered intravenous nitroglycerin via a PO-based tube had higher plasma concentrations of nitroglycerin and more significant reductions in blood pressure than those who received nitroglycerin through PVC- or PU-based tubes. This result suggests that the sorption of nitroglycerin is less in the PO-based tube than in the PVC- or PU-based tube, indicating that the tube materials used for drug administration may play a critical role in drug efficacy and safety.
